# Targeting Solid Lipid Nanoparticles with Anisamide for Docetaxel Delivery to Prostate Cancer: Preparation, Optimization, and *In-vitro* Evaluation

**DOI:** 10.22037/ijpr.2020.113436.14302

**Published:** 2021

**Authors:** Milad Jalilian, Katayoun Derakhshandeh, Masoumeh Kurd, Hussein Lashani

**Affiliations:** a *Department of Pharmaceutics, School of Pharmacy, Hamadan University of Medical Sciences, Hamadan, Iran. *; b *Medicinal Plants and Natural Products Research Center, Hamadan University of Medical Sciences, Hamadan, Iran. *; c *Pharmaceutical Nanotechnology Research Center (ZPNRC), Zanjan University of Medical Sciences, Zanjan, Iran.*

**Keywords:** Targeted drug delivery, Docetaxel, Solid lipid nanoparticles, Prostate cancer, Cytotoxicity

## Abstract

The purpose of the current study was to prepare and characterize the targeted solid lipid nanoparticles (SLNs) containing docetaxel (DTX) for prostate cancer treatment. The goal has been achieved by locating anisamide (Anis) ligand on the surface of SLNs, which can interact with the overexpressed sigma receptor on the prostate cancer cells. DTX loaded SLNs were prepared by high shear homogenization and ultra-sonication method and optimized by applying experimental design. The average particle size and the entrapment efficiency of the optimum DTX-SLN were 174 ± 9.1 nm and 83 ± 3.34%, respectively. The results of differential scanning calorimetry showed that DTX had been dispersed as amorphous in the nanocarriers. Scanning electron microscopy (SEM) images confirmed the nanoscale size and spherical shape of the nanoparticles. The cytotoxicity studies have demonstrated that IC_50_ of free drug, DTX-SLN and DTX-SLN-Anis was 0.25 ± 0.01, 0.23 ± 0.02, 0.12 ± 0.01 nM on PC3 cell line and 20.9 ± 3.89, 18.74 ± 7.43, and 14.68 ± 5.70 nM on HEK293 cell line, respectively. Targeted DTX-SLN-Anis was acted more effectively on prostate cancer cells in comparison to DTX-SLN and free drug. The results of this study have depicted that the anti-cancer drug loaded in targeted SLNs can be a promising way for cancer treatment. In addition, performing *in-vivo* studies will be complementary to these findings.

## Introduction

Prostate cancer (PCa) is one of the most prevalent male malignancies and the second leading cause of death by cancer in industrialized countries. The advancement of PCa not only means the long-range metastasis but also the development of the autonomic nerves into the tumour microenvironment. Adrenergic neurosignalling activates an angiogenic pathway, whereas parasympathetic nerves initiate cholinergic neurosignalling resulting in tumour dissemination and metastasis (1, 2).

Sigma receptors are well known membrane-bound proteins, which are considerably overexpressed on some human tumours, such as non-small cell lung carcinoma, melanoma, breast tumours, and PCa (3-6).

Some benzamide derivatives have shown a high affinity to sigma receptors. Due to this issue, application of the sigma-receptor binding ligands has been suggested for the diagnosis and targeted therapy of a variety of tumours including PCa (4, 7).

The focus here is on anisamide, a low-molecular-weight benzamide derivative used as a tumor-directing moiety in functionalized nano systems, based on its alleged interaction with sigma receptors (8). Docetaxel (DTX) is an adjuvant chemotherapy drug widely used for multiple solid tumours treatment; Although DTX has many advantages in cancer therapy, it can cause serious side effects, such as neutropenia, myelosuppression, anemia, and hypersensitivity reaction, which restrict its clinical applications (9). DTX is a semisynthetic diterpene extracted from leaves of the European yew, *Tuxus baccata* (10). The structure of DTX resembles another antitumor diterpene, paclitaxel, which is extracted from the Pacific yew Tuxus brevifoliu. These compounds are mitotic spindle poisons that promote tubulin polymerization and inhibit depolymerisation of microtubules (11, 12). With the progress of nanotechnology and its applications in medicine, nanoparticle drug delivery systems can revive the clinical potential of abandoned compounds by reducing their toxicity. Solid lipid nanoparticles (SLNs) are a class of lipid-based nanoparticles with low toxicity and high stability compared to liposomal and polymeric nanoparticles, which can effectively control the rate of drug release and act as an excellent nanocarrier for drug targeting (13).

Therefore, the main objective of the current study was to design and prepare optimized DTX-SLN-Anis nanoparticles as an efficient drug delivery system for PCa treatment (14). Moreover, antiprolifrative efficacy of the most promising nanoparticles has been evaluated using intracellular cytotoxicity tests on prostate cancer cell line (PC3) and human embryonic kidney cells HEK293.

## Experimental

*Materials*

The PC3 and HEK293 cells were purchased from the Pasteur Institute of Iran. Stearic acid, tween 80, spermine, 4-methoxybenzoyl chloride, dichloro- methane, triethylamine, DTX, phosphate buffered saline (PBS), dimethylsulfoxide (DMSO), MTT (3-(4, 5- Dimethylthiazol-2-yl)-2, 5-diphenyltetrazolium Bromide), and all other reagents have purchased from Merck and Sigma-Aldrich.

*Preparation of DTX loaded SLN nanoparticles*

*Synthesis of Spermine- Anisamide*

**Figure 1:**
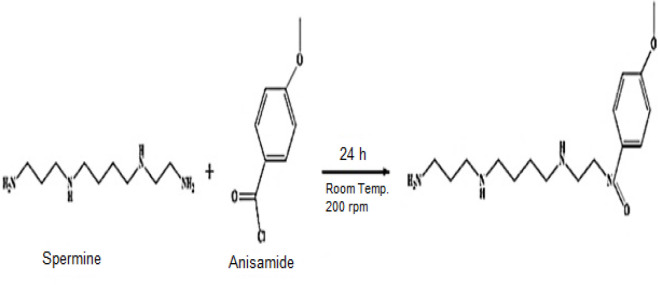
Schematic representation of spermine- Anis conjugate synthesis pathway

4-methoxybenzoyl chloride, anisamide, (200 mg, 1.176 mmol) was dissolved in 10 mL dichloromethane. Spermine (50 μL, 0.227 mmol) and triethylamine (50 μL, 0.36 mmol) were added to the solution, and the mixture was stirred at 200 *×g* for 24 h at room temperature. The solvent was removed via rotary evaporation Figure 1 (15).

*Preparation of SLN-DTX-Anis nanoparticles*

SLNs were prepared by the altered high shear homogenization and ultrasonication method. Stearic acid (50 mg) was melted at about 80 °C, and certain amounts of DTX (2 mg) and spermine-Anis (100 mg) was added to obtain a clear solution. The aqueous phase was prepared by dissolving 70 mg of tween 80 in 15 mL of distilled water and heated at 85 °C (above the melting temperature of the lipid phase). The lipid phase was then gradually added to the aqueous phase, and the mixture was homogenized at 15000 *×g* for 10 min. The obtained nano dispersion was sonicated for 5 min and cooled down under stirring conditions of 300 *×g* and at room temperature for 10 min. Eventually, the prepared nanoparticles have been lyophilized and stored at 4 ºC (16, 17).

*Optimization of nanoparticle preparation method*

In order to keep the number of experiments affordable for optimization of the nanoparticles preparation method, the central composite design was chosen in Design-Expert software (trial version, 7.0.0). Three formulation parameters were selected as the independent variables, including the amount of drug (DTX), stearic acid, and tween 80. These factors are presented in Table 1. The measured dependent variables were the size of the nanoparticles, polydispersity index (PDI), zeta potential, and entrapment efficiency (EE). Finally, an optimized formulation was selected, and other characterization parameters have been evaluated. The performance of nanoparticles is affected by many parameters, mainly size, shape, surface charge, and toxicity of the particles.

*Nanoparticle characterization*

*Particle size and surface morphology*

Nanoparticle’s mean diameter and polydispersity measurements were performed by photon correlation spectroscopy (PCS) and surface charge of the nanoparticle by electrophoretic movement using a Malvern Zetasizer nano series system (ZEN3600 model, Malvern Instruments, Worcestershire, England). The operation was performed at the temperature of 25 °C, a medium refractive index of 1.33, and viscosity of 0.8872 cP with 90degree light scattering. The applied samples have been appropriately diluted with deionized water. Each measurement has been performed in triplicate at room temperature. Results are presented as mean ± standard deviation.

The morphology of nanoparticles has been determined using scanning electron microscopy (SEM) (Phillips, the Netherlands). In order to make the samples conductive, they were coated with a thin layer of gold before the results were recorded by SEM at 20 kV.

*FT-IR characterization of nanoparticles*

Fourier transformed-infrared spectroscopy (FT-IR) spectra were obtained using (Bruker FTIR spectrometer). FT-IR identifies chemical bonds in a molecule by producing an infrared absorption spectrum. To consider the FT-IR spectrum of DTX, Blank SLN, DTX-SLN-anis, spermine, and anisamide, 2 mg of the samples were mixed with 10 mg of KBr and compressed into tablets. The IR spectra of these tablets were obtained in an absorbance mode and in the spectral region of 450 to 4,000 cm^−1^.

*Differential scanning calorimetry (DSC) analysis*

Thermal analysis of DTX- SLN- Anis nanoparticles and bulk materials has been performed using Differential Scanning Calorimetry (DSC) (Malvern, England). About 4 mg of dried samples were completely sealed in the aluminum pans. Air was considered as a reference (Empty pan). Samples were heated from 30 to 200 °C (heating rate of 10 °C/min) under the Ar atmosphere.

*Drug loading (DL) and entrapment efficiency (EE) determination*

Drug loading (DL) and EE% were calculated by measuring the amount of unloaded DTX. Briefly, 20 mL of the freshly prepared nanodispersion was centrifuged at 30000 *×g* for 20 min using ultracentrifuge in order to separate free DTX aqueous solution. The free DTX concentrations were analyzed by the UV-VIS method at a wavelength of 229 nm. The percentages of DL and EE have been determined by applying the Equations 1 and 2 (18-21):

EE (%) = (Weight of DTX in nanoparticle"s " )/(Drug total weight) × 100% 

Equation 1.

DL (%) = (Weight of DTX in nanoparticles)/(Total nanoparticle weight) × 100% 

Equation 2.

*In-vitro drug release*

*In-vitro* release studies of DTX from DTX-SLN and DTX-SLN-Anis were investigated using the dialysis method (cut off = 12000 Da).

DTX has low solubility in water (6-7 µg/mL); therefore, sink conditions were maintained for release studies by phosphate buffer solution (pH 7.4) as the release medium (22). The drug-loaded nanoparticles of a volume equivalent to 10 mg DTX were dialyzed against receptor medium over a period of 48 h. In order to mimic the biological conditions, a bio incubator (Heidolph, Germany) was used at the temperature of 37 °C and the rate of 100 cycles/min. The amount of released DTX in the receptor medium has been determined by applying the spectrophotometer method, and the accumulated release profile versus time was drawn. This study was performed with three replicates, and the outcomes were explained as mean values ± standard deviation (23).

*Release kinetics*

Zero-order, First-order, Hixson–Crowell, Higuchi, and Korsmeyer–Peppas models were selected to fit on the release profile. The goodness of fitting for the release kinetic models was evaluated with correlation coefficient values (*R*^2^).

*In-vitro cytotoxicity assay*

PC3 and HEK293 cell lines were used to evaluate the cytotoxicity of targeted nanoparticles. Cells were cultured in the RPMI1640 medium containing 10% FBS and placed in a humidified incubator at 37 °C with 5% CO_2_ overnight. Cells were seeded in 100 μL of growth medium using 96-well culture microplates at a density of 10^4 ^per well. After 24 h of incubation, cells were treated with different concentrations (0.062-1 nM) of DTX-SLN, DTX-SLN-Anis, anisamide, blank SLN, and DTX. The cells treated with a cytotoxic drug and the untreated cells were used as the positive, negative, and control, respectively, and the plain medium as the blank. At specified time intervals, the medium was removed, and 100 μL of DMSO was added.

The absorbance of cells has been measured by using a microplate reader scanning spectrophotometer at 570 nm. Relative cell viability was calculated using Equation 3:

Viability (%) = (absorbance of each wells)/(average absorbance of untreated wells) × 100% 

Equation 3.

 The half-maximal inhibitory concentration (IC_50_) was also calculated by using the diagram of viability percentage (*y*-axis) and log *C *(*x*-axis) Made by Graph Pad Prism 6 software (24-26).

## Results and Discussion

SLN has been introduced as a new drug delivery system since the early 1990s in order to replace liposomes, emulsions, and polymer nanoparticles.

SLNs have several benefits, including biodegradability, biocompatibility, increasing the physicochemical stability of the drug, controlling its release and desirable pharmacokinetic properties. In addition, there is no need to use organic solvents in their manufacturing process and they can be easily produced and sterilized on a large scale (27).

To date, many efforts and significant advances in DTX delivery have been made as an effective drug for cancer treatment.

*Preparation and optimization of SLN-DTX-Anis nanoparticles*

SLNs were produced based on the prescribed method. The runs are shown in Table 1. Each run was repeated three times, and the results were obtained as mean ± SD for dependent quantities. The report of an optimal formulation is also shown in Table 1. Particle size, PDI, zeta potential, and percentage of drug entrapment of this optimal formulation are also presented.

*DTX-SLN-Anis nanoparticles characteristics*

The mean particle size and distribution width of different formulations were measured and the values are shown in Table 1. The results were 237 nm and 0.45 for DTX-SLN and 174 nm and 0.22 for DTX-SLN-Anis respectively.

Due to the results, the DTX-SLN-Anis has shown a narrow distribution width and considerable small particle size. The possible reason can be attributed to the steric hinderance hydrophilic legand (anisamide) which prevents aggregation of particles during initial steps of preparation (28). Carrier size is determined by the molecular geometry of ligands on its surface. The hydrophobic core of the carrier is protected from the aqueous environment by the hydrophilic external shell. The more the hydrophobic part is in contact with the aqueous environment, the more the carrier structure loses integrity (29, 30).

**Figure 2:**
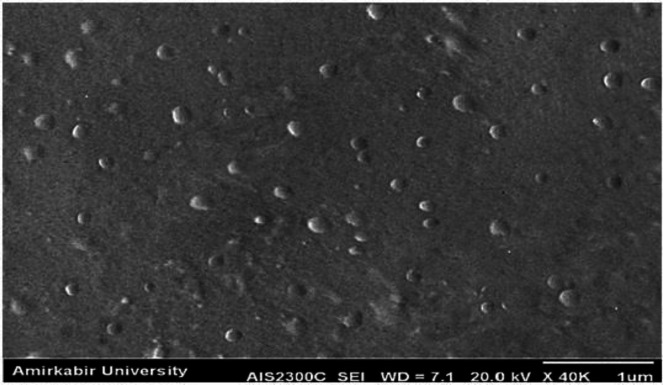
SEM image of SLN -DTX- Anis nanoparticles

The SEM image of the optimum DTX-SLN-Anis formulation is shown in Figure 2. This image illustrates that the investigated particles had a round shape and homogeneous particle size distribution, approximately similar to Zetasizer report.

**Figure 3:**
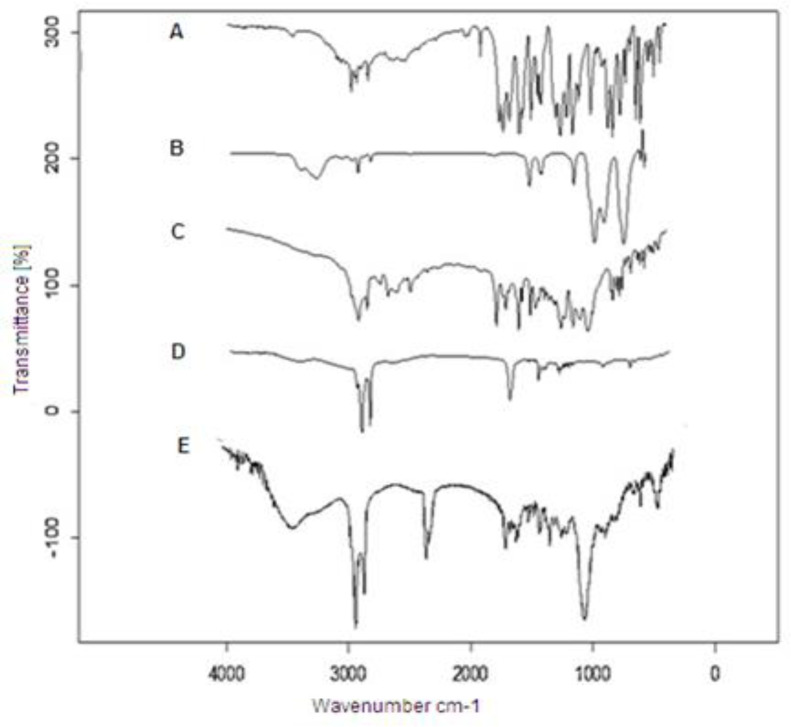
FT-IR spectra of (A) Anisamide, (B) Spermine, (C) DTX-SLN-Anis, (D) Blank SLN and (E) DTX

Figure 3 illustrates the FTIR spectra of DTX, spermine, anisamide, DTX-SLN, and DTX-SLN- Anis. The FTIR spectrum of DTX shows the characteristic band at 3421 cm^-1^ for -N-H stretching of alkanes. The Peak at 1734 cm^-1^ is assigned to -C-O- stretching vibrations, and the peak at 1647 cm^-1^ is assigned to -N-H plane bending vibration. The FTIR spectrum of Anis shows a characteristic band at 3060 cm^-1^Ar–C–H stretching, 1032 cm^-1^Ar–C=O stretching, and 764 and 834 cm^-1^ C–H.

Comparison of the obtained data with the SLN-DTX-Anis spectrum exhibits the presence of DTX and Anis characteristic peaks in the spectrum of SLN-DTX-Anis, which demonstrates the successful loading of DTX and the presence of Anis in the nanoparticles. These results show a good agreement with earlier studies (31, 32).

**Figure 4:**
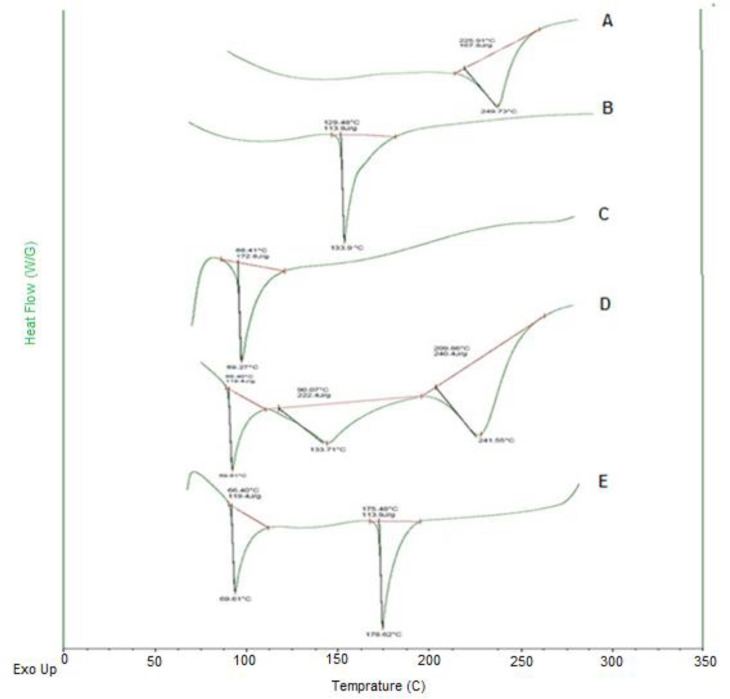
DSC spectra (A) DTX, (B) Anisamide, (C) SLN, (D) the physical mixture DTX, SLN and Anisamide and (E) DTX-SLN-Anis

Differential scanning calorimetric of blank SLN, DTX-SLN, DTX-SLN-Anis, and the physical mixture of formulation materials are displayed in Figure 4. The thermogram of DTX demonstrated a sharp endothermic peak at 225 °C that corresponds to its melting point (T_m_). It can be related to the change in the physical state of the entrapped drug in the lipid core of nanoparticles.

The thermogram of SLNs demonstrated a sharp endothermic peak at 69 °C that corresponded to the stearic acid melting point (T_m_) and confirmed the solid-state of the lipid within the SLN (33).

The thermogram of anisamide demonstrated a sharp endothermic peak at 133.7 °C, which corresponds to its melting point (T_m_). The peak of Anis in the DTX-SLN-Anis showed a shift from 133.7 to 176.6 °C, which can be related to the covalent bond of anisamide molecules at the surface of nanoparticles.

*In-vitro release*

**Figure 5:**
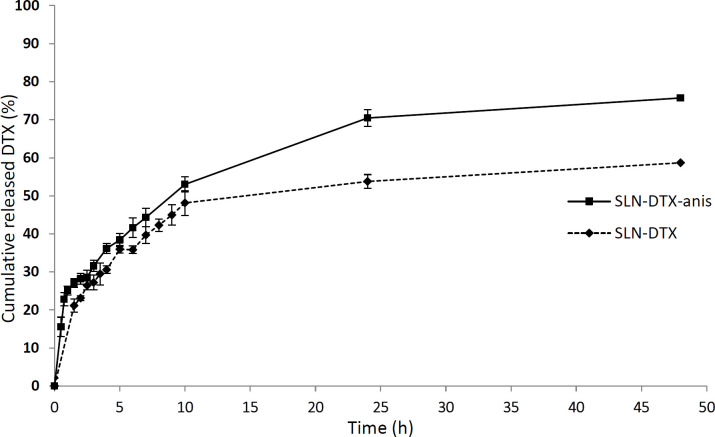
*The release profiles of* *DTX from* *SLN- DTX-Anis and SLN -DTX - behavior through the dialysis membrane in PBS (pH 7.4, 37 ºC).*

The release profiles of the drug from DTX-SLN and DTX-SLN-Anis are demonstrated in Figure 5.

In both formulations, an initial burst release has been observed due to the accumulation of DTX on the surface layer of the nanoparticles. In the second step, a steady and sustained release was observed. This phenomenon has been reported in some studies about nanocarriers (34). After about 50 h, approximately 57 and 76% of DTX was released from DTX-SLN and DTX-SLN-Anis, respectively. The higher rate of drug release in DTX-SLN-Anis can be related to the presence of hydrophilic groups of anisamide in the outer layer of the nanoparticle. This layer grows over time as more water penetrates into the nanocarrier . Also, *smaller* particles of DTX-SLN-Anis have a larger surface area-to-volume ratio; therefore, a higher release level was observed (29, 35).

The various kinetic models were fitted to the release data of DTX from prepared nanoparticles (Table 2). Based on the correlation coefficient criteria, the release profiles of DTX loaded in nanoparticles were best fitted on the Higuchi model (R^2 ^= 0.93). This model is based on the hypotheses that drug diffusion takes place only in one and matrix swelling and dissolution are negligible. It describes the drug release as a diffusion process based on Fick’s laws, which is square root time dependent (25).

*In-vitro cytotoxicity*

In the current study, the cytotoxicity studies of prepared nano-formulations have been performed on PC3 and HEK-293 cell lines.

**Figure 6:**
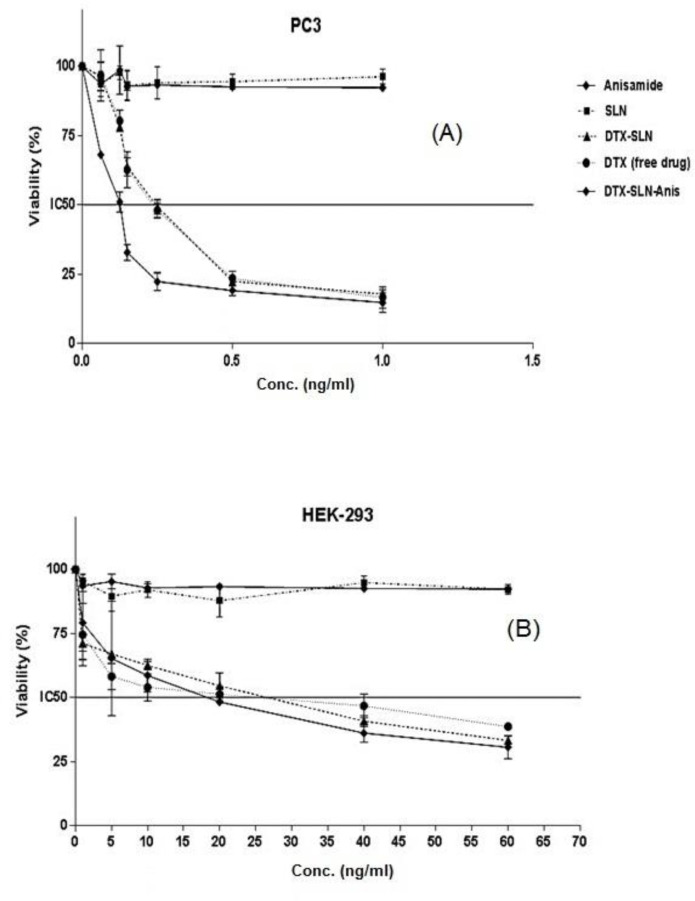
Results of MTT Test and IC_50_ values comparison. (A) PC3 Cell Line, (B) HEK-293 cell line

The results of cytotoxicity on PC3 cells (Figure 6a) were shown that IC_50_ of 0.25 ± 0.01, 0.23 ± 0.02, 0.12 ± 0.01 for DTX, DfX-SLN, DTX-SLN-Anis, respectively. Blank SLN and Anis have not shown remarkable cytotoxicity on PC3 cells. In addition, the results of cytotoxicity on HEK-293 cells were shown that IC_50_ of DTX, DTX-SLN, and DTX-SLN-Anis were 20.9 ± 3.89, 18.74 ± 7.43, and 14.68 ± 5.70, respectively. SLNs and Anis have not shown any remarkable cytotoxicity on HEK-293 cells (Figure 6b).

The statistical test, which is based on the results of the studies on cytotoxicity, has shown a high cytotoxic effect of DTX-SLN-Anis on the cells. Generally, it can be concluded that drug entrapment in the nanoparticles will apply higher toxicity on the cells in comparison to the free drugs.

Moreover, the results of cytotoxicity studies on the HEK293 were shown that the targeted formulation has a lower IC_50_ than the other nanocarriers and free drugs , which could be due to the presence and overexpression of the sigma-1 receptor on these cells (15). The results have been in good accordance with other reports on this targeting ligand and nanocarrier.

The study of Banerjee and coworkers has demonstrated the use of small molecular weight ligands for efficient targeting of drugs loaded in liposomal nano carriers to the sigma receptor, expressed by prostate cancer cells both *in-vitro* and *in-vivo* conditions (36).

Anisamide conjugated lyotropic nano-liquid crystalline particles were designed as a smart optical carrier for tumor imaging and therapy (31). Urandur and coworkers have developed the inverse hexagonal nano-liquid crystalline particles that are able to host formononetin, a phytoestrogen with known anticancer activity, and tetraphenylethene, an iconic optical beacon with aggregation-induced emission signature, simultaneously.

*In-vitro* and *in-vivo* studies supported the enhanced efficacy of targeted nanoparticles in comparison to nontargeted nanoparticles and free drugs (37).

Xu et al prepared DTX-loaded hepatoma-targeted SLNs with galactosylated dioleoyl phosphatidyl ethanolamine for the treatment of locally advanced and metastatic human hepatocellular carcinoma. This carrier has shown better antitumor efficacy and reduced toxicity compared with Taxotere in a murine model bearing hepatoma (38).

**Table 1:** Experimental design matrix and analysis of variance

**Run**	** Tween 80 (mg)**	** Lipid (mg)**	** Drug (mg)**	** Ligand (mg)**	**Size (nm)**	**PDI**	**Zeta (mV)**		**EE (%)**
1	100	70	1	50	371 ± 32.2	0.35 ± 0.002	(-6) ± 2.31		85 ± 4.11
2	70	70	1	50	352 ± 27.1	0.48 ± 0.001	(-27) ± 3.24		80 ± 3.23
3	40	70	1	50	431 ± 12.51	0.41 ± 0.004	(-22) ± 4.11		83 ± 5.11
4	100	50	1	50	282 ± 15.22	0.24 ± 0.005	(-10) ± 5.22		79 ± 6.42
5	70	50	1	50	390 ± 17.1	0.37 ± 0.001	(-13) ± 3.16		74 ± 3.64
6	40	50	1	50	563 ± 22.3	0.36 ± 0.005	(-18) ± 4.17		71 ± 7.56
7	100	30	1	50	632 ± 13.5	0.53 ± 0.003	(-19) ± 4.76		63 ± 5.44
8	70	30	1	50	840 ± 22.7	0.62 ± 0.002	(-16) ± 5.26		66 ± 2.33
9	40	30	1	50	242 ± 11.3	0.23 ± 0.001	(-20) ± 2.33		68 ± 5.88
10	100	70	2	50	227 ± 16.4	0.27 ± 0.001	(-23) ± 3.25		79 ± 3.29
11	70	70	2	50	536 ± 24.1	0.48 ± 0.002	(-22) ± 3.16		86 ± 6.12
12	40	70	2	50	472 ± 19.4	0.35 ± 0.001	(-20) ± 4.67		83 ± 2.18
13	100	50	2	50	394 ± 16.4	0.38 ± 0.002	(-16) ± 5.21		76 ± 3.64
14	70	50	2	50	174 ± 9.1	0.22 ± 0.001	(-22) ± 2.13		83 ± 3.34
15	40	50	2	50	423 ± 11.2	0.41 ± 0.001	(-13) ± 2.19		76 ± 3.77
16	100	30	2	50	736 ± 25.3	0.52 ± 0.001	(-17) ± 4.54		58 ± 4.66
17	70	30	2	50	283 ± 22.1	0.28 ± 0.002	(-11) ± 2.13		63 ± 3.51
18	40	30	2	50	243 ± 11.7	0.31 ± 0.005	(-12) ± 4.81		71 ± 2.22
**Runs of preparation and Optimization of SLNs**									
**Tween(mg)**	** Lipid(mg)**	** Drug(mg)**	** Ligand(mg)**	**Size (nm)**	**PDI**	**Zeta(mv)**		**EE (%)**	
70	50	2	50	174 ± 9.1	0.22 ± 0.001	(-22) ± 2.13		83 ± 3.34	

**Table 2:** Summary of model parameters of DTX release data

**Models**	**RSQ**	**Slope**	**Intercept**
Zero order	0.7523	0.02	30.08
First order	0.8688	-0.0002	1.8523
Higuchi	0.9325	1.2980	15.702
Korsmeyer–Peppas	0.7182	0.4321	0.5419
Hixson–Crowell	0.8339	0.0007	0.7228

## Conclusion

Prostate cancer is one of the most common cancers among the male population. One of the possible treatments is the use of chemotherapy drugs. Due to the severe side effects of chemotherapeutics, a lot of studies have concentrated on the new drug delivery systems recently.

Applying nanoparticles as the carriers to realize passive and active targeting can improve the efficacy of chemotherapy medicines significantly, reduce the mortality rate of cancer patients, and improve the life quality of the patients.

Anisamide derivative ligand has shown the high affinity for sigma receptors. It was hypothesized that incorporating it into the SLNs containing DTX can specifically target and deliver the drug to prostate cancer cells that overexpressed sigma receptors.

SLNs were prepared by high shear homogenization method. The size, PDI, zeta potential and EE of the prepared nanocarriers have been studied and the results were acceptable.

The drug release pattern of the nanoparticles follows the Higuchi model. The prepared SLNs have been tested on 2 cell lines in order to evaluate the cytotoxicity effects. The results have shown the notable cytotoxicity effect on PC3 cell line. However, this effect was not considerable on HEK293.

According to the obtained results and by comparing the groups with each other, it can be concluded that the conjugation of SLNs with anisamide can improve the treatment efficacy in comparison to DTX and SLN-DTX carrier. In this way the drug dose and the side effects can be reduced in cancer treatment.

However, there is not any commercially available nanoformulation of DTX in the market currently. Due to this, further research is required for commercializing DTX nanoparticles.

## References

[1] Petrylak DP, Tangen CM, Hussain MH, Lara Jr PN, Jones JA, Taplin ME, Burch PA, Berry D, Moinpour C, Kohli M, Benson MC, Small EJ, Raghavan D, Crawford ED (2004). Docetaxel and estramustine compared with mitoxantrone and prednisone for advanced refractory prostate cancer. N. Eng. J. Med..

[2] March B, Faulkner S, Jobling P, Steigler A, Blatt A, Denham J, Hondermarck H (2020). Tumour innervation and neurosignalling in prostate cancer. Nat. Rev. Urol..

[3] Vilner BJ, John CS, Bowen WD (1995). Sigma-1 and sigma-2 receptors are expressed in a wide variety of human and rodent tumor cell lines. Cancer Res..

[4] John CS, Bowen WD, Fisher SJ, Lim BB, Geyer BC, Vilner BJ, Wahl RL (1999). Synthesis, in vitro pharmacologic characterization, and preclinical evaluation of N-[2-(1′-piperidinyl) ethyl]-3-[125I] iodo-4-methoxybenzamide (P [125I] MBA) for imaging breast cancer. Nucl. Med. Biol..

[5] John CS, Vilner BJ, Bowen WD (1994). Synthesis and Characterization of -N-(N-Benzylpiperidin-4-yl)-4-iodobenzamide, a New sigma Receptor Radiopharmaceutical: High-Affinity Binding to MCF-7 Breast Tumor Cells. J. Med. Chem..

[6] Hou C, Tu Z, Mach R, Kung HF, Kung MP (2006). Characterization of a novel iodinated sigma-2 receptor ligand as a cell proliferation marker. Nucl. Med. Biol..

[7] John CS, Vilner BJ, Geyer BC, Moody T, Bowen WD (1999). Targeting sigma receptor-binding benzamides as in-vivo diagnostic and therapeutic agents for human prostate tumors. Cancer Res..

[8] Dasargyri A, Kümin CD, Leroux JC (2017). Targeting nanocarriers with anisamide: fact or artifact? Adv. Mater..

[9] Petrylak DP (2006). The treatment of hormone-refractory prostate cancer: docetaxel and beyond. Rev. Urol..

[10] Ringel I, Horwitz SB (1991). Studies with RP 56976 (taxotere): a semisynthetic analogue of taxol. J. Natl. Cancer Inst..

[11] Diaz JF, Andreu JM (1993). Assembly of purified GDP-tubulin into microtubules induced by taxol and taxotere: reversibility, ligand stoichiometry, and competition. Biochemistry.

[12] Schiff PB, Horwitz SB (1980). Taxol stabilizes microtubules in mouse fibroblast cells. Proc. Natl. Acad. Sci..

[13] Sadegh Malvajerd S, Azadi A, Izadi Z, Kurd M, Dara T, Dibaei M, Sharifzadeh M, Akbari Javar H, Hamidi M (2018). Brain delivery of curcumin using solid lipid nanoparticles and nanostructured lipid carriers: Preparation, optimization, and pharmacokinetic evaluation. ACS Chem. Neurosci..

[14] Omwoyo WN, Ogutu B, Oloo F, Swai H, Kalombo L, Melariri P, Maroa Mahanga G, Waweru Gathirwa J (2014). Preparation, characterization, and optimization of primaquine-loaded solid lipid nanoparticles. Int. J. Nanomedicine.

[15] Dasargyri A, Hervella P, Christiansen A, Proulx ST, Detmar M, Leroux JC (2016). Findings questioning the involvement of Sigma-1 receptor in the uptake of anisamide-decorated particles. J. Control. Release.

[16] Bhatt S, Sharma J, Singh M, Saini V (2018). Solid lipid nanoparticles: a promising technology for delivery of poorly water-soluble drugs. Acta Pharm. Sci..

[17] Derakhshandeh K, Hosseinalizadeh A, Nikmohammadi M (2011). The effects of PLGA microparticles on intestinal absorption of p-glycoprotein substrate using the everted rat intestinal sac model. Arch. Pharmacal. Res..

[18] Perez MH, Zinutti C, Lamprecht A, Ubrich N, Astier A, Hoffman M, Bodmeier R, Maincent P (2000). The preparation and evaluation of poly (ε-caprolactone) microparticles containing both a lipophilic and a hydrophilic drug. J. Control. Release.

[19] Sánchez E, Moreno A, Vicent M, Salvador M, Bonache V, Klyatskina E, Santacruz I, Moreno R (2010). Preparation and spray drying of Al2O3–TiO2 nanoparticle suspensions to obtain nanostructured coatings by APS. Surf. Coat. Tech..

[20] Smith G (2010). Bioanalytical method validation: notable points in the 2009 draft EMA Guideline and differences with the 2001 FDA Guidance. Bioanalysis..

[21] Ahmadi F, Derakhshandeh K, Jalalizadeh A, Mostafaie A, Hosseinzadeh L (2015). Encapsulation in PLGA-PEG enhances 9-nitro-camptothecin cytotoxicity to human ovarian carcinoma cell line through apoptosis pathway. Res. Pharm. Sci..

[22] Lee E, Kim H, Lee IH, Jon S (2009). In-vivo antitumor effects of chitosan-conjugated docetaxel after oral administration. J. Control. Release.

[23] Owuor JJ, Oloo F, Ouma D, Omwoyo WN (2017). Optimization and Characterization of PrimaquineLoaded solid lipid nanoparticles (SLN) for liver schinonticide targeting by freeze drying. Drug Des. Dev. Ther..

[24] Mosallaei N, Jaafari MR, Hanafi-Bojd MY, Golmohammadzadeh S, Malaekeh-Nikouei B (2013). Docetaxel-loaded solid lipid nanoparticles: preparation, characterization, in vitro, and in-vivo evaluations. J. Pharm. Sci..

[25] Bijari N, Ghobadi S, Derakhshandeh K (2017). Irinotecan binds to the internal cavity of beta-lactoglobulin: A multi-spectroscopic and computational investigation. J. Pharm. Biomed. Anal..

[26] Saghafi Z, Mohammadi M, Mahboobian MM, Derakhshandeh K (2021). Preparation, characterization, and in-vivo evaluation of perphenazine-loaded nanostructured lipid carriers for oral bioavailability improvement. Drug Dev. Ind. Pharm..

[27] Zhang L, Zhang N (2013). How nanotechnology can enhance docetaxel therapy. Int. J. Nanomedicine.

[28] Müller RH, Jacobs C, Kayser O (2001). Nanosuspensions as particulate drug formulations in therapy: rationale for development and what we can expect for the future. Adv. Drug Deliv. Rev..

[29] Ling D, Hackett MJ, Hyeon T (2014). Surface ligands in synthesis, modification, assembly and biomedical applications of nanoparticles. Nano Today.

[30] Owen SC, Chan DP, Shoichet MS (2012). Polymeric micelle stability. Nano Today.

[31] Pawar H, Surapaneni SK, Tikoo K, Singh C, Burman R, Gill MS, Suresh S (2016). Folic acid functionalized long-circulating co-encapsulated docetaxel and curcumin solid lipid nanoparticles: in-vitro evaluation, pharmacokinetic and biodistribution in rats. Drug Deliv..

[32] Bell S, Subhashini S (2018). Self-assembled Chitosan-g-Poly (itaconic acid) nanoparticles: a potential drug carrier for docetaxel. Asian J. Chem..

[33] Oktay B (2018). Preparation of stearic acid/graphene oxide based form-stable composite phase change materials. Marmara Fen Bilimleri Dergisi.

[34] Aboutaleb E, Atyabi F, Khoshayand MR, Vatanara AR, Ostad SN, Kobarfard F, Dinarvand R (2014). Improved brain delivery of vincristine using dextran sulfate complex solid lipid nanoparticles: optimization and in-vivo evaluation. J. Biomed. Mater. Res..

[35] Ghori MU, Conway BR (2015). Hydrophilic matrices for oral control drug delivery. Am. J. Pharmacol. Sci..

[36] Banerjee R, Tyagi P, Li S, Huang L (2004). Anisamide-targeted stealth liposomes: a potent carrier for targeting doxorubicin to human prostate cancer cells. Int. J. Cancer.

[37] Urandur S, Banala VT, Shukla RP, Mittapelly N, Pandey G, Kalleti N, Mitra K, Rath SK, Trivedi R, Ramarao P and Mishra PR (2018). Anisamide-anchored lyotropic nano-liquid crystalline particles with AIE effect: a smart optical beacon for tumor imaging and therapy. ACS Appl. Mater. Interfaces.

[38] Xu Z, Chen L, Gu W, Gao Y, Lin L, Zhang Z, Xi Y, Li Y (2009). The performance of docetaxel-loaded solid lipid nanoparticles targeted to hepatocellular carcinoma. Biomaterials.

